# Supplementary figures and images for: Preliminary characterisation of the spatial immune and vascular environment in triple negative basal breast carcinomas using multiplex fluorescent immunohistochemistry
Source: PLoS One. 2025 Jan 10;20(1):e0317331. doi: 10.1371/journal.pone.0317331 (PMC11723538; doi:10.1371/journal.pone.0317331)

**S1 Fig. Tissue segmentation using HALO software**

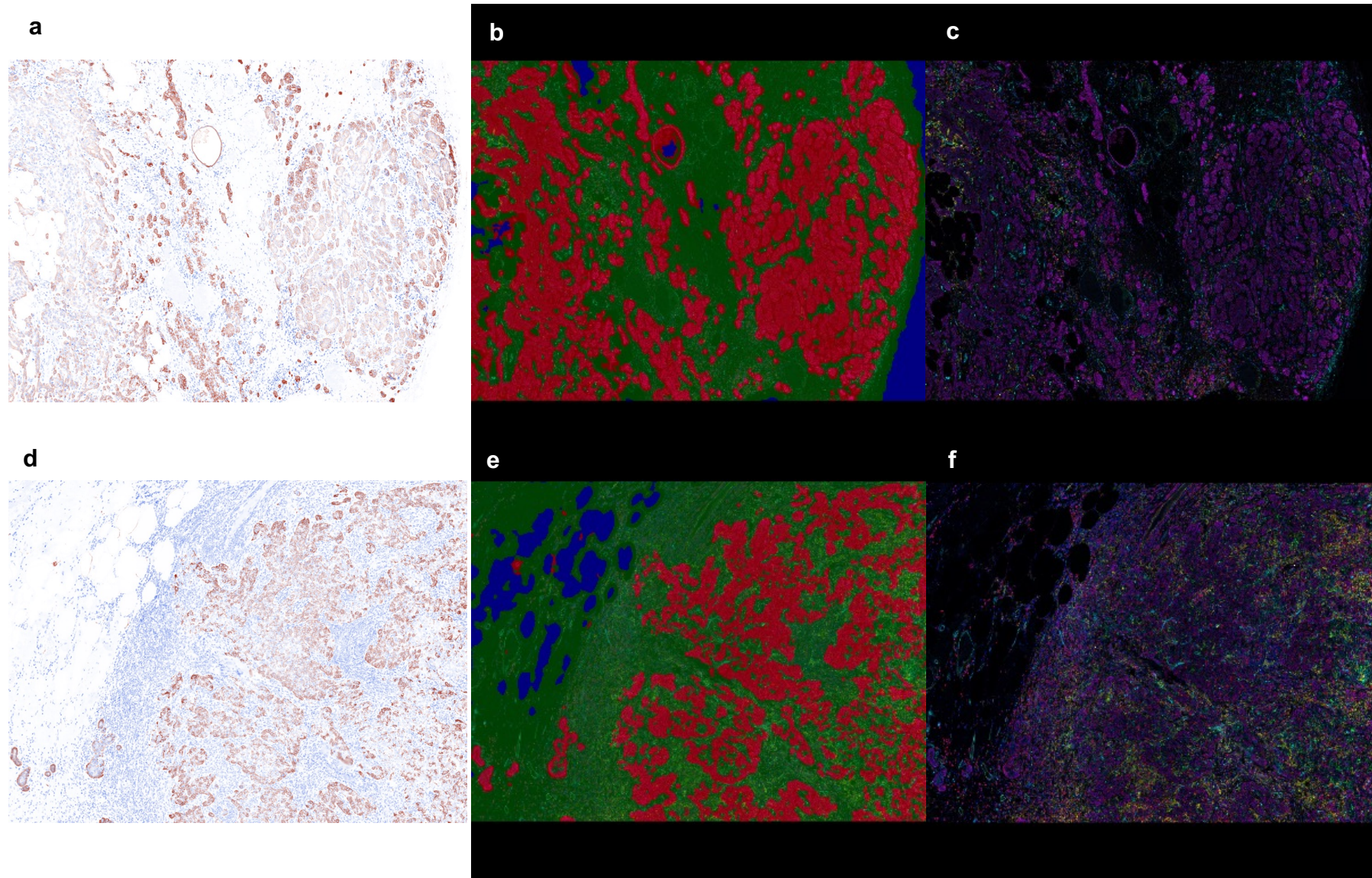

Supplement: S1 Fig — a) and d) tumour compartment defined by the positive expression of the pan-cytokeratin marker visualised by DAB, b) and e) the corresponding same tumour compartment (red) together with stroma (green) and other (blue) defined by HALO software, c) and f) merged mIF images. (PDF) [file pone.0317331.s001.pdf]

**S2 Fig. Specific cell phenotyping using HALO software.**

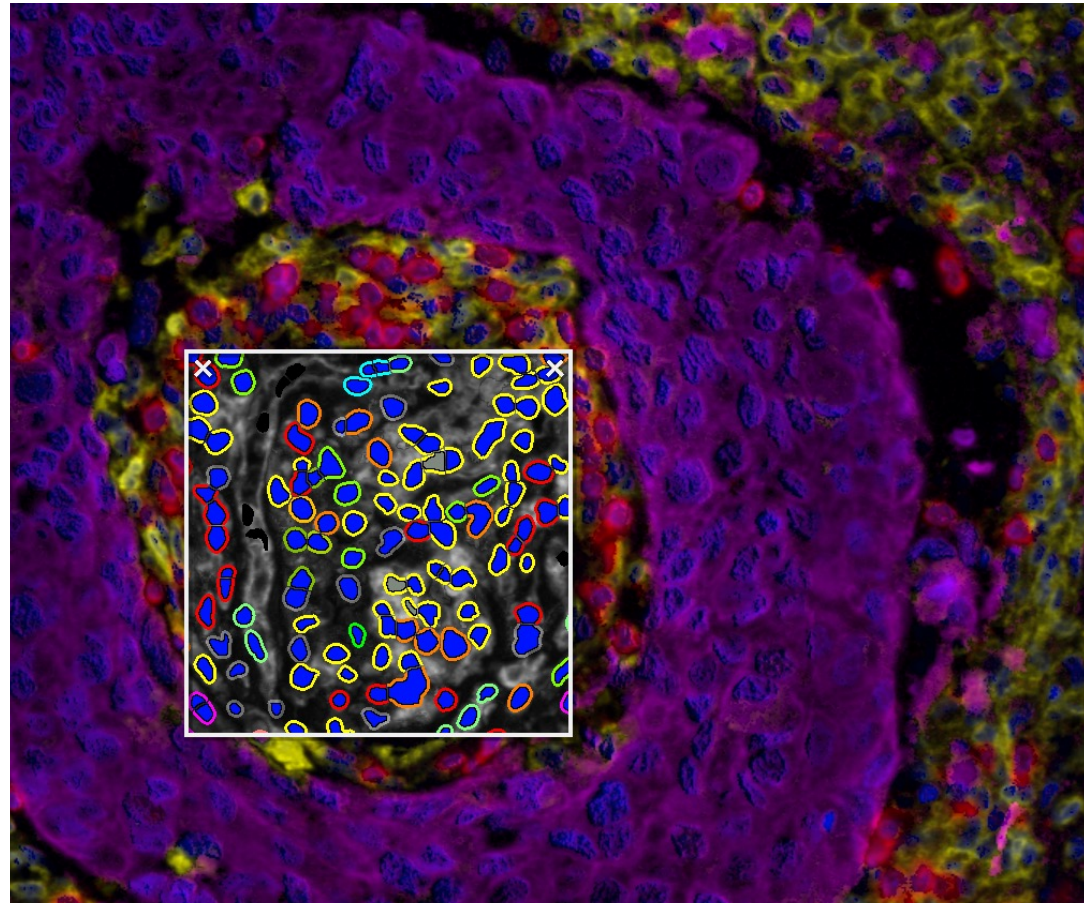

Supplement: S2 Fig — Cell phenotype showing in the ‘real-time view window’ (the square frame). The real-time view window and HALO software confirms as blue the DAPI signal in each nucleus from the raw IF image, and again the HALO software applies different coloured rings around nuclei, delineating the multiplex co-localisation of multiple markers and enabling specific cell phenotyping. (PDF) [file pone.0317331.s002.pdf]
