# Supplementary material for: Preliminary characterisation of the spatial immune and vascular environment in triple negative basal breast carcinomas using multiplex fluorescent immunohistochemistry
Source: PLoS One. 2025 Jan 10;20(1):e0317331. doi: 10.1371/journal.pone.0317331 (PMC11723538; doi:10.1371/journal.pone.0317331)

S3 Fig. Zonal compartmentalisation for T cell infiltration analysis around BVs using HALO software.

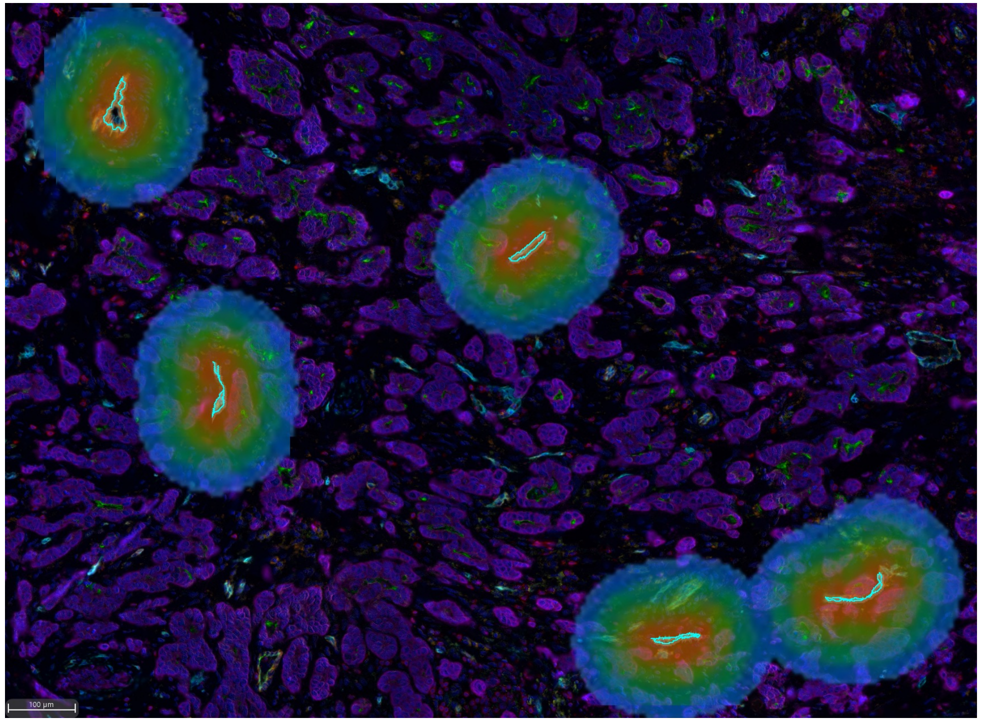

Supplement: S3 Fig — CD3+ density (cells/mm2) up to 120μm from each blood vessel consisting of 4 bands of 30um indicated by the coloured bands (red, yellow, green, blue), is measured collectively for each BV phenotype using the HALO Spatial Analysis module. (PDF) [file pone.0317331.s003.pdf]
