# Supplementary material for: Preliminary characterisation of the spatial immune and vascular environment in triple negative basal breast carcinomas using multiplex fluorescent immunohistochemistry
Source: PLoS One. 2025 Jan 10;20(1):e0317331. doi: 10.1371/journal.pone.0317331 (PMC11723538; doi:10.1371/journal.pone.0317331)

S4 Fig. CD3$^+$ T cells in tumour compartment and stromal compartment displaying their activation markers.

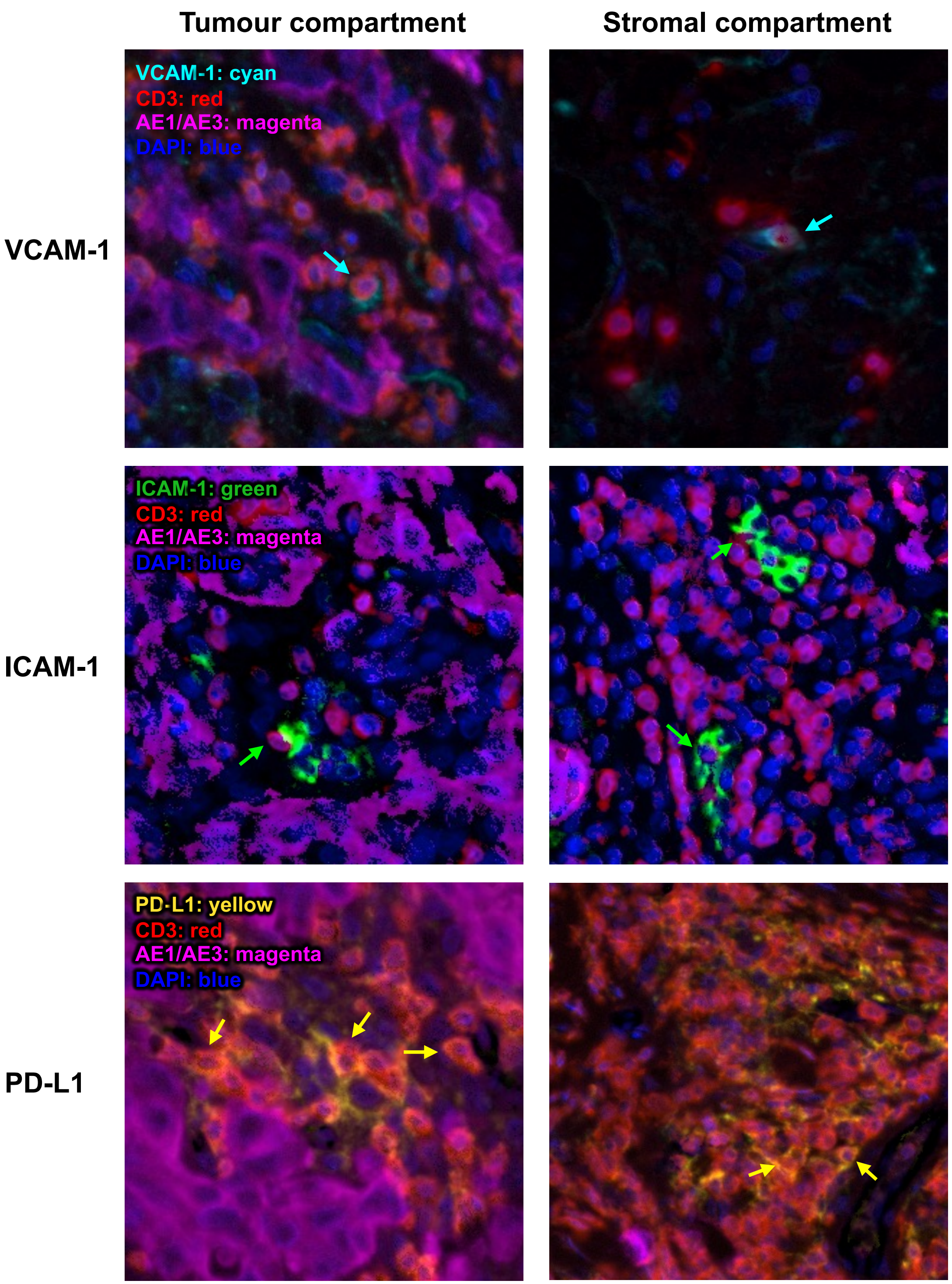

Supplement: S4 Fig — (PDF) [file pone.0317331.s004.pdf]
