# Supplementary material for: Preliminary characterisation of the spatial immune and vascular environment in triple negative basal breast carcinomas using multiplex fluorescent immunohistochemistry
Source: PLoS One. 2025 Jan 10;20(1):e0317331. doi: 10.1371/journal.pone.0317331 (PMC11723538; doi:10.1371/journal.pone.0317331)

S5 Fig. CD3⁺ T cell densities shown in each 30μm-width zone with 30μm increments; 0-30μm, 30-60μm, 60-90μm, 90-120μm from BVs, revealing high to low T cell gradient around ICAM-1⁺ BVs from proximal to distal.

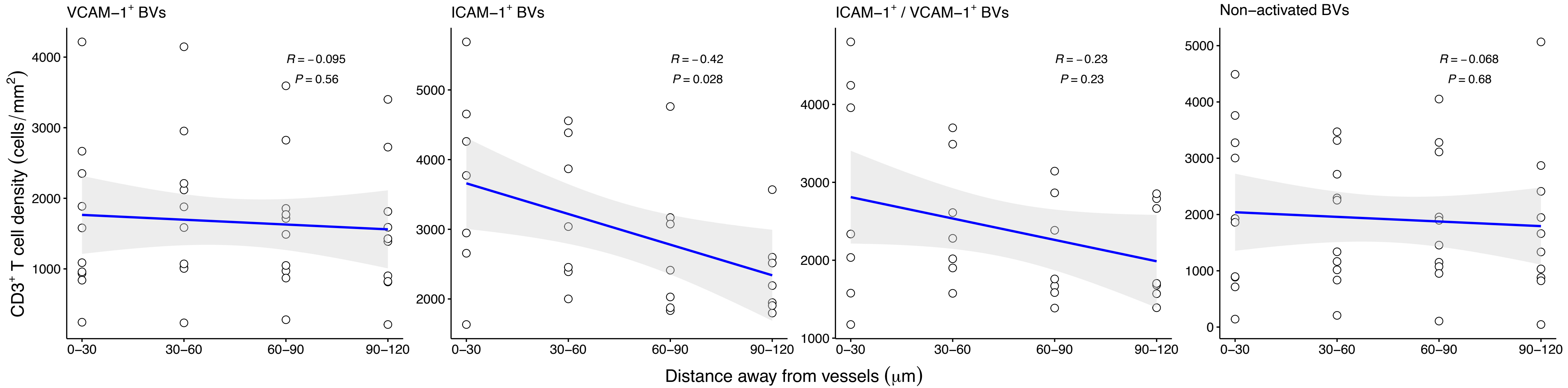

Supplement: S5 Fig — The distinctive T cell gradient exclusive to ICAM-1+ BVs, which was evidently absent in any other types of activated or non-activated BVs. It was supported by a significant negative correlation (Spearman correlation R = -0.42, P = 0.028). R specifies the Spearman correlation, the line indicates the regression line and the grey shaded area specifies standard error in the graphs. (PDF) [file pone.0317331.s005.pdf]
