# Supplementary material for: Preliminary characterisation of the spatial immune and vascular environment in triple negative basal breast carcinomas using multiplex fluorescent immunohistochemistry
Source: PLoS One. 2025 Jan 10;20(1):e0317331. doi: 10.1371/journal.pone.0317331 (PMC11723538; doi:10.1371/journal.pone.0317331)

S6 Fig. Statistical analysis showing the significant association between basal subtype and 'upregulated' gene cluster.

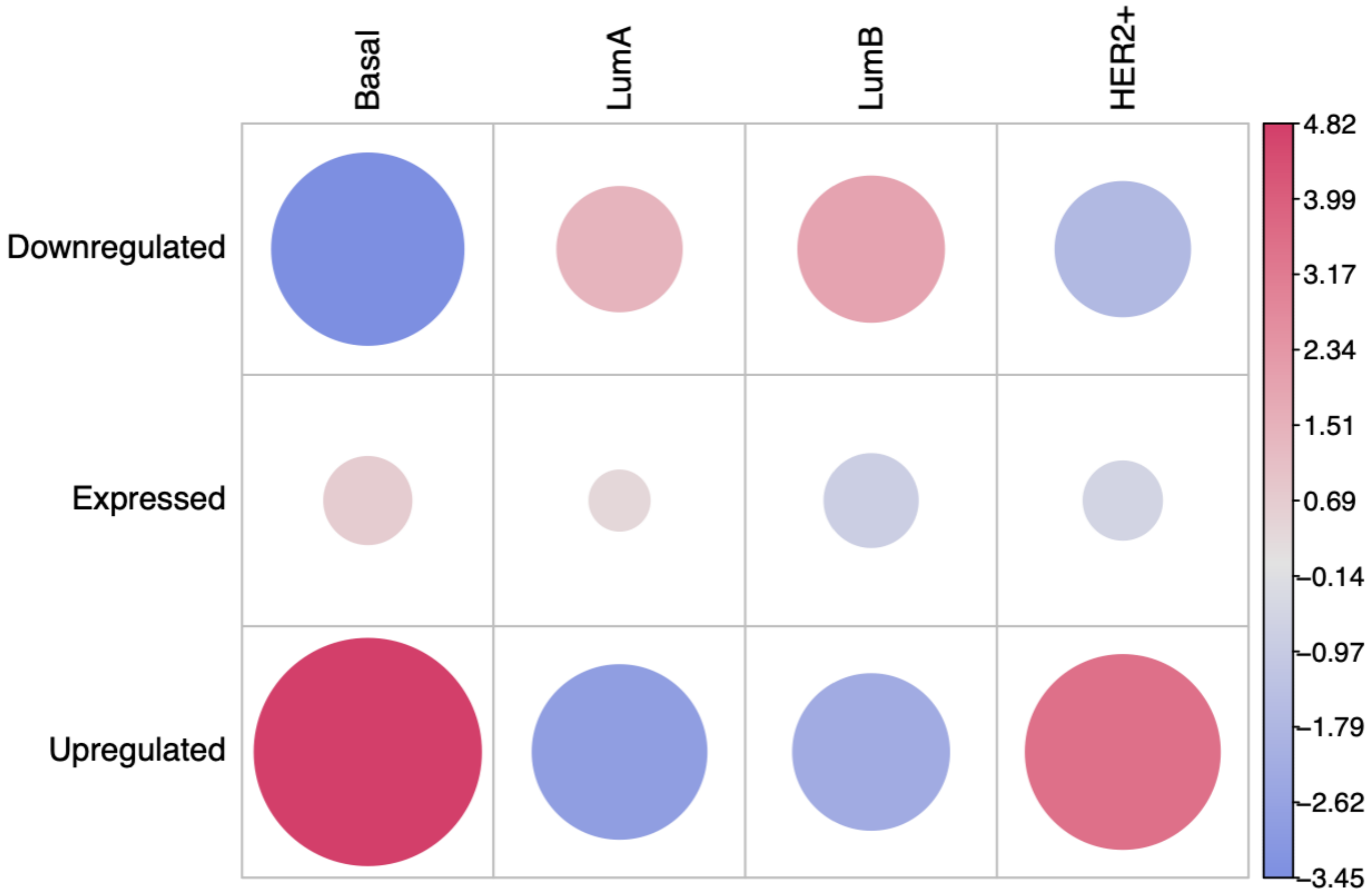

Supplement: S6 Fig — Depiction of Pearson residuals (r2) by cluster and PAM50 subtype, derived from the Chi-square test of independence. The size of the circle is proportional to the residual’s contribution. Red indicates a positive association, and blue indicates a negative association. (PDF) [file pone.0317331.s006.pdf]
