# Supplementary material for: Preliminary characterisation of the spatial immune and vascular environment in triple negative basal breast carcinomas using multiplex fluorescent immunohistochemistry
Source: PLoS One. 2025 Jan 10;20(1):e0317331. doi: 10.1371/journal.pone.0317331 (PMC11723538; doi:10.1371/journal.pone.0317331)
Supplement: S1 File — (DOCX) [file pone.0317331.s008.docx]

**Characterising the spatial immune and vascular environment of triple negative basal breast carcinomas by multiplex fluorescent immunohistochemistry.**

**Short title**

**The immune and vascular environment of triple negative basal breast carcinomas**

Elena A. Takano^1*^, Metta K. Jana^2^, Luis E. Lara Gonzalez^3,4^, Jia-Min B. Pang^1^, Roberto Salgado^3,5^, Sherene Loi^3,4^ & Stephen B. Fox^1,4*^

^1^ Department of Pathology, Peter MacCallum Cancer Centre, Melbourne, Victoria, Australia

^2^ Centre for Advanced Histology and Microscopy, Peter MacCallum Cancer Centre, Melbourne, Victoria, Australia

^3^ Cancer Research Division, Peter MacCallum Cancer Centre, Melbourne, Victoria, Australia

^4^ The Sir Peter MacCallum Department of Oncology, The University of Melbourne, Parkville, Victoria, Australia

^5^ GZA-ZNA-Hospitals, Antwerp, Belgium

* Corresponding authors

Email: elena.takano@petermac.org (EAT)

Email: stephen.fox@petermac.org (SBF)

# **Supporting information**

## **Methods**

### **mIF**

4μm thick FFPE sections from each case and respective control tissues were mounted onto coated slides and baked for 45 minutes at 60 ^o^C prior to de-waxing and hydration to water. The initial antigen retrieval was carried out in a pressure cooker (Pascal, Dako Cytomation California Inc., USA) in a retrieval buffer, 1x AR6 buffer (PerkinElmer), which is an appropriate buffer for the first antibody in the sequence (CD3 antibody) at 124 ^o^C with pressure set at 15 PSI for 3 minutes. Endogenous peroxidase activity in the tissue was quenched with 3% hydrogen peroxide diluted in PBS for 5 minutes at room temperature. Primary antibodies, HRP conjugated secondary antibodies, and TSA Opal fluorophores were incubated at the condition listed in Table 1. Between each staining rounds, an antigen retrieval step was carried out in a microwave to remove the bound primary-secondary antibody complex from the tissue antigens, allowing for the introduction of the subsequent primary antibody. Spectral DAPI solution provided in the kit was applied to the multiplexed sections for 5 minutes at room temperature after the final microwave step ensuring the removal of the antibody complex. All the stained slides were then cover slipped in an aqueous anti-fade mounting medium (Citifluor, Electron Microscopy Sciences, USA).

### **Segmentation of tumour compartment and cell types**

Segmentation of the tissue was performed using the multiplex markers (S1 Fig.). The tumour cell compartment was defined using morphology and the pan-cytokeratin marker, AE1/AE3. The inverse areas containing DAPI positive cells with negative for pan-cytokeratin marker were defined as stroma. The ‘other’ classification was applied for the area where no tissues were detected. The number of the cells for each cell type were based on their respective staining patterns (S2 Fig.). For example, the cells with DAPI positive nuclei define the total number of cells, and other markers in the panel assist with defining the marker positive cytoplasmic/membrane stained cells. The results were expressed as the number of marker positive cells and density (number of marker positive cells/mm^2^) in each compartment, tumour and stroma.
