# Supplementary material for: Preliminary characterisation of the spatial immune and vascular environment in triple negative basal breast carcinomas using multiplex fluorescent immunohistochemistry
Source: PLoS One. 2025 Jan 10;20(1):e0317331. doi: 10.1371/journal.pone.0317331 (PMC11723538; doi:10.1371/journal.pone.0317331)
Supplement: Attachment — Submitted filename: Response to Reviewers_Dec24.docx [file pone.0317331.s010.docx]

We would like to thank both reviewers once again for their time and effort in evaluating the manuscript. We are deeply appreciative. Please refer to our responses in blue, addressing each comment.

Reviewer's Responses to Questions

Comments to the Authors:
1. If the authors have adequately addressed your comments raised in a previous round of review and you feel that this manuscript is now acceptable for publication, you may indicate that here to bypass the “Comments to the Author” section, enter your conflict of interest statement in the “Confidential to Editor” section, and submit your "Accept" recommendation.

Reviewer #1: (No Response)

Reviewer #2: All comments have been addressed

Thank you.

2. Is the manuscript technically sound, and do the data support the conclusions?

The manuscript must describe a technically sound piece of scientific research with data that supports the conclusions. Experiments must have been conducted rigorously, with appropriate controls, replication, and sample sizes. The conclusions must be drawn appropriately based on the data presented.

Reviewer #1: Yes

Reviewer #2: Partly

The design of the study was to use a highly selected discovery cohort that had been chosen to cover specific ranges of immune infiltration within which then could be validated in a larger independent *in silico* cohort. This discovery cohort revealed relationships between CD3+T cells and vessels, an observation confirmed by the *in silico* analysis thus supporting our hypotheses. We of the opinion that study design with additional strong *in silico* analysis are sufficient to support the findings in the study.

3. Has the statistical analysis been performed appropriately and rigorously?

Reviewer #1: Yes

Reviewer #2: Yes

Thank you.

4. Have the authors made all data underlying the findings in their manuscript fully available?

The [PLOS Data policy](http://track.editorialmanager.com/CL0/http:%2F%2Fwww.plosone.org%2Fstatic%2Fpolicies.action%23sharing/1/010f01938a1f6cc1-a0645138-4f67-4b5b-90e5-518ed0133070-000000/mhiEexNM41DEnBXqgrp_OU7H-kSdFr3EGR-pTnlZOGI=187) requires authors to make all data underlying the findings described in their manuscript fully available without restriction, with rare exception (please refer to the Data Availability Statement in the manuscript PDF file). The data should be provided as part of the manuscript or its supporting information, or deposited to a public repository. For example, in addition to summary statistics, the data points behind means, medians and variance measures should be available. If there are restrictions on publicly sharing data—e.g. participant privacy or use of data from a third party—those must be specified.

Reviewer #1: No

All of our graphs are Box and Whisker Plots and visually displaying the data points distribution through their quartiles with upper and lower extremes. We also included representative images throughout in the manuscript. Although we could potentially upload all the scanned mIF images, we believe that the data referred to in the question pertains more to genomic/quantitative information rather than images and trust this is the case. Nevertheless, we would be happy to upload images on advice from the Editor. Please let us know how we can address this. Thank you.

Reviewer #2: Yes

5. Is the manuscript presented in an intelligible fashion and written in standard English?

PLOS ONE does not copyedit accepted manuscripts, so the language in submitted articles must be clear, correct, and unambiguous. Any typographical or grammatical errors should be corrected at revision, so please note any specific errors here.

Reviewer #1: Yes

Reviewer #2: Yes

Thank you.

6. Review Comments to the Author

Please use the space provided to explain your answers to the questions above. You may also include additional comments for the author, including concerns about dual publication, research ethics, or publication ethics. (Please upload your review as an attachment if it exceeds 20,000 characters)

**Reviewer #1:**

Dear authors,
Thank you for addressing my comments so extensively. I think this has taken the manuscript to a higher level. Especially the incorporation of images in the manuscript makes it much more insightful and appealing.

Thank you, we agree that the paper is much stronger.

I only have some minor comments left:

Line 69: now all other subtypes have been removed, but they are for importance in figure 7. I think it would help the reader if you at least mention the other subtypes of TNBC here as well.

Thank you for your suggestion. We added the other subgroups and now it reads “TNBC is a heterogeneous group of tumours that can be classified further into subtypes based on their morphology and transcriptomic profile [2]. The most common is basal-like with other groups being claudin-low, mesenchymal, luminal androgen receptor and immunomodulatory.”

Line 78-81: here is is stated that PD-L1 expression in TNBC is mostly on the tumor-infiltrating lymphocyte (TIL) compartment. This is an incorrect statement. When reviewing reference 13 (ref 14 may be removed because it is a podcast) it is stated that “PD-L1 expression on tumor-infiltrating immune cells”. Tumor-infiltrating immune cells and lymphocytes are not terms that are mutually exclusive. In general, PD-L1 is scored on either the tumor compartment or immune cell compartment. You can’t determine if the PD-L1 is on lymphocytes or on other immune cells. Please adjust this.

We agree that immune cells are not synonymous with lymphocytes. However, most PD-L1 positive immune cells in TNBCs are lymphocytes and from personal scoring of hundreds of breast cancers for PD-L1, it is the lymphocytes that predominantly stain. Nevertheless, we have changed to use immune cells for ease.

Line 223: It would be nice if this sentence refers to a new figure 1a-b where there is a multiplex image of T cells in the tumor and stromal compartment and then continue on for the next subnumbers with the quantification.

We hope our understanding on your suggestion is correct. Line 223 in both versions of our revised manuscript (with track changes and clean version) belongs to the subsection “*in silico* analysis of tumour microenvironment on the TCGA breast cancer data” and “Statistical analysis” respectively. Hence we don’t see how these paragraphs we can relate to Fig 1. Nevertheless, we have interpreted this to add a figure, which we have done to supplementary information -S4.Fig (our preference) of CD3 T cells in tumour compartment and stromal compartment with the activated T cells in these compartments.

Figure 1: I noticed that there has not yet been made a nicely fitting lay-out for figure 1. I am not sure if this is conform PLOS ONE policy. Figure 1a, b, c and d are uploaded separately and not as one larger figure 1. It would have been nicer for the reviewing process to also be able to comment on this part.

Apologies for not yet creating a multi-panel figure. This is because the figure preparation checklist states at the very top that "PLOS ONE waives all formatting requirements until your manuscript has received a provisional Editorial Acceptance decision.” However, we have now created a multi-panel figure.

Figure 2: The resolution of these images is quite bad again. There is also sort of a white box in the image, which is not clearly visible. It would be more clearly if next to these composite images, also black and white images are shown of the cells of interest as has been done in this publication (/doi.org/10.1002/eji.202350616) in figure 3.

Apologies for the poor resolution. As recommended in the PLOS ONE checklist, we used the PACE tool to correct/reduce the file size before uploading. The white boxes around the cells of interest were generated by the HALO software. We acknowledge that the boxes appear faint however, they are designed to highlight the cell phenotypes without causing obstruction therefore, to aid in identifying the boxes, arrows were added as indicators. However, we have now made the boxes drawn with thicker lines. Also please refer to the new montages (bottom row), displaying a single channel with DAPI, for each of the corresponding panel d), e) and f).

Figure 3: also see if you can represent the co-expression in the example that is given for figure 2.

We think co-expression of 2 markers on BVs (BVs are made up with multiple endothelial cells expressing different adhesion molecules) are clearly visible in this figure.

Next to that, the examples of figure 3 can be merged with it quantification of figure 4.

We have made a multi-panel figure, merging figure 3 with our quantification figure 4 as your suggestion.

Figure 7: resolution is quite bad.

(Now Fig 6)

We apologize again for the poor resolution. However, we have followed the PLOS ONE figure guidelines and used the PACE tool for verification. Please refer to the newly created figure, which is now available in a larger file with improved resolution.

**Reviewer #2:**

Dear authors thanks for your response. While data from 10 cases can be valuable for exploratory purposes or hypothesis generation, the authors should frame their conclusions as preliminary and avoid making broad claims. I would recommend emphasizing the need for further studies with larger sample sizes to validate these results and strengthen the robustness of the findings. I suggest modifying the title as "Preliminary Characterization of the Spatial Immune and Vascular Microenvironment in Triple Negative Basal Breast Carcinoma Using Multiplex Fluorescent Immunohistochemistry"

Thank you for your suggestion, we have changed the title as per your suggestion and have emphasised in the summary that validation of our findings is needed “It will be important to validate these findings in an independent cohort.”
